# Social mobility and biological aging among older adults in the United States

**DOI:** 10.1093/pnasnexus/pgac029

**Published:** 2022-03-29

**Authors:** Gloria Huei-Jong Graf, Yalu Zhang, Benjamin W Domingue, Kathleen Mullan Harris, Meeraj Kothari, Dayoon Kwon, Peter Muennig, Daniel W Belsky

**Affiliations:** Department of Epidemiology, Columbia University Mailman School of Public Health, New York, NY 10032, USA; Robert N Butler Columbia Aging Center, Columbia University Mailman School of Public Health, New York, NY 10032, USA; Columbia School of Social Work, New York, NY 10027, USA; Peking University Institute of Population Research, Beijing, China; Stanford Graduate School of Education, Stanford, CA 94305, USA; Department of Sociology, Carolina Population Center, University of North Carolina at Chapel Hill, Chapel Hill, NC 27599, USA; Robert N Butler Columbia Aging Center, Columbia University Mailman School of Public Health, New York, NY 10032, USA; Robert N Butler Columbia Aging Center, Columbia University Mailman School of Public Health, New York, NY 10032, USA; UCLA Fielding School of Public Health, Department of Epidemiology, Los Angeles, CA 90095, USA; Department of Health Policy and Management, Columbia University Mailman School of Public Health, New York, NY 10032, USA; Department of Epidemiology, Columbia University Mailman School of Public Health, New York, NY 10032, USA; Robert N Butler Columbia Aging Center, Columbia University Mailman School of Public Health, New York, NY 10032, USA

**Keywords:** Social Mobility, Biological Aging, Aging, Social Determinants of Health, Health Disparities, Life Course

## Abstract

Lower socioeconomic status is associated with faster biological aging, the gradual and progressive decline in system integrity that accumulates with advancing age. Efforts to promote upward social mobility may, therefore, extend healthy lifespan. However, recent studies suggest that upward mobility may also have biological costs related to the stresses of crossing social boundaries. We tested associations of life-course social mobility with biological aging using data from participants in the 2016 Health and Retirement Study (HRS) Venous Blood Study who provided blood-chemistry (*n* = 9,255) and/or DNA methylation (DNAm) data (*n* = 3,976). We quantified social mobility from childhood to later-life using data on childhood family characteristics, educational attainment, and wealth accumulation. We quantified biological aging using 3 DNAm “clocks” and 3 blood-chemistry algorithms. We observed substantial social mobility among study participants. Those who achieved upward mobility exhibited less-advanced and slower biological aging. Associations of upward mobility with less-advanced and slower aging were consistent for blood-chemistry and DNAm measures of biological aging, and were similar for men and women and for Black and White Americans (Pearson-r effect-sizes ∼0.2 for blood-chemistry measures and the DNAm GrimAge clock and DunedinPoAm pace-of-aging measures; effect-sizes were smaller for the DNAm PhenoAge clock). Analysis restricted to educational mobility suggested differential effects by racial identity; mediating links between educational mobility and healthy aging may be disrupted by structural racism. In contrast, mobility producing accumulation of wealth appeared to benefit White and Black Americans equally, suggesting economic intervention to reduce wealth inequality may have potential to heal disparities in healthy aging.

Significance Statement Upward social mobility may disrupt effects of early-life disadvantage on aging-related health decline. However, the stresses of crossing social boundaries can have biological costs. To investigate the balance of these forces, we analyzed social mobility from reports of childhood circumstances, education, and later-life wealth in 9,286 older adults in the US HRS. We quantified life-course health impacts of social mobility from blood-chemistry and DNAm analysis of biological aging. We found that educational mobility alone benefited Black Americans less than White Americans, whereas mobility that produced accumulation of wealth into later-life was associated with delayed biological aging across social categories. Black–White disparities in healthy-aging outcomes of educational mobility may reflect inequalities in social gains realized from education.

## Introduction

Children who grow up poor get sick and die younger than their peers who grow up in more socioeconomically advantaged families (1, 2). This inequality is mediated by a range of chronic diseases and health problems that become more frequent as individuals age, suggesting that childhood disadvantage may actually accelerate the aging process (3). Breakthroughs in aging biology have revealed a set of molecular changes that accumulate as individuals grow older, undermining resilience and driving vulnerability to multiple different chronic diseases, disability, and mortality (4). While there is currently no gold standard to measure this progressive loss of system integrity, several methods have been proposed (5). Current state-of-the-art methods are algorithms that combine information from multiple clinical or genomic measurements to track changes that occur in peoples’ bodies as they age. In longitudinal studies that track children through midlife, these algorithm-based methods reveal that people who grew up in disadvantaged households are biologically older and are aging more rapidly as adults as compared to peers with more advantaged childhoods, and vice versa (6–10). In cross-sectional studies, children and adults in higher socioeconomic-status households exhibit less-advanced and slower biological aging as compared to those with lower socioeconomic status (11, 12). These findings suggest that upward socioeconomic mobility, in which children climb the social ladder to achieve higher levels of status attainment than their family of origin, may interrupt processes that accelerate aging.

Conversely, upward mobility may also have biological costs. The stresses of climbing the social ladder, such as prolonged, high-effort coping, can damage health (13–17). This effect may be especially pronounced for groups facing structural barriers to upward mobility, such as Black Americans. If upward mobility accelerates biological aging, then interventions to build opportunity for at-risk children will need to devise additional strategies to offset potential health costs.

We tested if life-course socioeconomic mobility was associated with slower or faster biological aging in a national sample of US adults, the US Health and Retirement Study (HRS). We quantified social mobility from childhood to later-life based on retrospective reports by participants about their childhood socioeconomic conditions and structured interviews about household wealth. We quantified biological aging using DNA methylation (DNAm)- and physiology-based methods. Our analysis proceeded in 3 steps. We first tested how life-course socioeconomic disadvantage was associated with accelerated biological aging. We next tested whether upward social mobility was associated with blunting or amplification of associations between early-life socioeconomic disadvantage and accelerated biological aging. Finally, we tested if associations of mobility with biological aging were consistent for men and women and for Black and White adults to evaluate the hypothesis that the cost of social mobility could be more pronounced for groups who face structural barriers to upward mobility. We conducted parallel analysis of participants’ educational mobility.

## Methods

### Data and participants

We analyzed data from participants in the 2016 HRS who provided blood-chemistry and DNAm data in the Venous Blood Study (VBS). The HRS is a nationally representative longitudinal survey of US residents ≥ 50 years of age and their spouses (https://hrs.isr.umich.edu/documentation). The HRS has been fielded every 2 years since 1992. A new cohort of 51–56-y-olds and their spouses is enrolled every 6 years to maintain representativeness of the US population over 50 years of age. Participants are asked about 4 broad areas: income and wealth; health, cognition, and use of healthcare services; work and retirement; and family connections. As of the 2016 data release, the HRS included data collected from 42,515 individuals in 26,600 households. The 2016 VBS collected biomarker data from a subset of HRS participants who consented to a venous blood draw, as part of a larger effort to understand biological mechanisms linking social factors and health (*n* = 9,286). DNAm assays were done on a nonrandom subsample of VBS participants representative of the larger HRS sample (*n* = 3,989). We linked HRS data curated by the RAND Corporation with new data collected as part of the HRS's 2016 VBS ((18, 19). Our final analytic sample included all individuals who (1) participated in the 2016 wave of the HRS, (2) provided biomarker and/or DNAm data through the VBS, and (3) provided retrospective reports of socioeconomic indicators in childhood, middle adulthood, and later-life. The final analytic sample was 9,255 for analyses using biomarker measures of biological aging and 3,976 for analyses using DNAm measures of biological aging. Comparison of VBS participants to the full HRS is reported in Table S1 (Supplementary Material; Panel A).

### Measures

#### Biological aging

Biological aging is the gradual and progressive decline in system integrity that occurs with advancing chronological age, mediating aging-related disease and disability (20). While there is no gold standard measure of biological aging (5), current state-of-the-art methods use machine learning to sift through large numbers of candidate biomarkers and parameterize algorithms that predict aging-related parameters, including chronological age, mortality risk, and rate of decline in system integrity. Algorithms are developed in reference datasets and can then be applied to new datasets to test hypotheses.

For our analysis, we selected 3 blood-chemistry measures and 3 DNAm measures of biological aging shown in the previous work to predict morbidity and mortality (6, 21–25), and which also demonstrated more advanced or more rapid aging in low socioeconomic status adults (6, 7, 26, 27). We compared different measures of biological aging to evaluate robustness of findings and to compare the sensitivity of blood-chemistry and DNAm biological-aging algorithms.

**Fig. 1.**
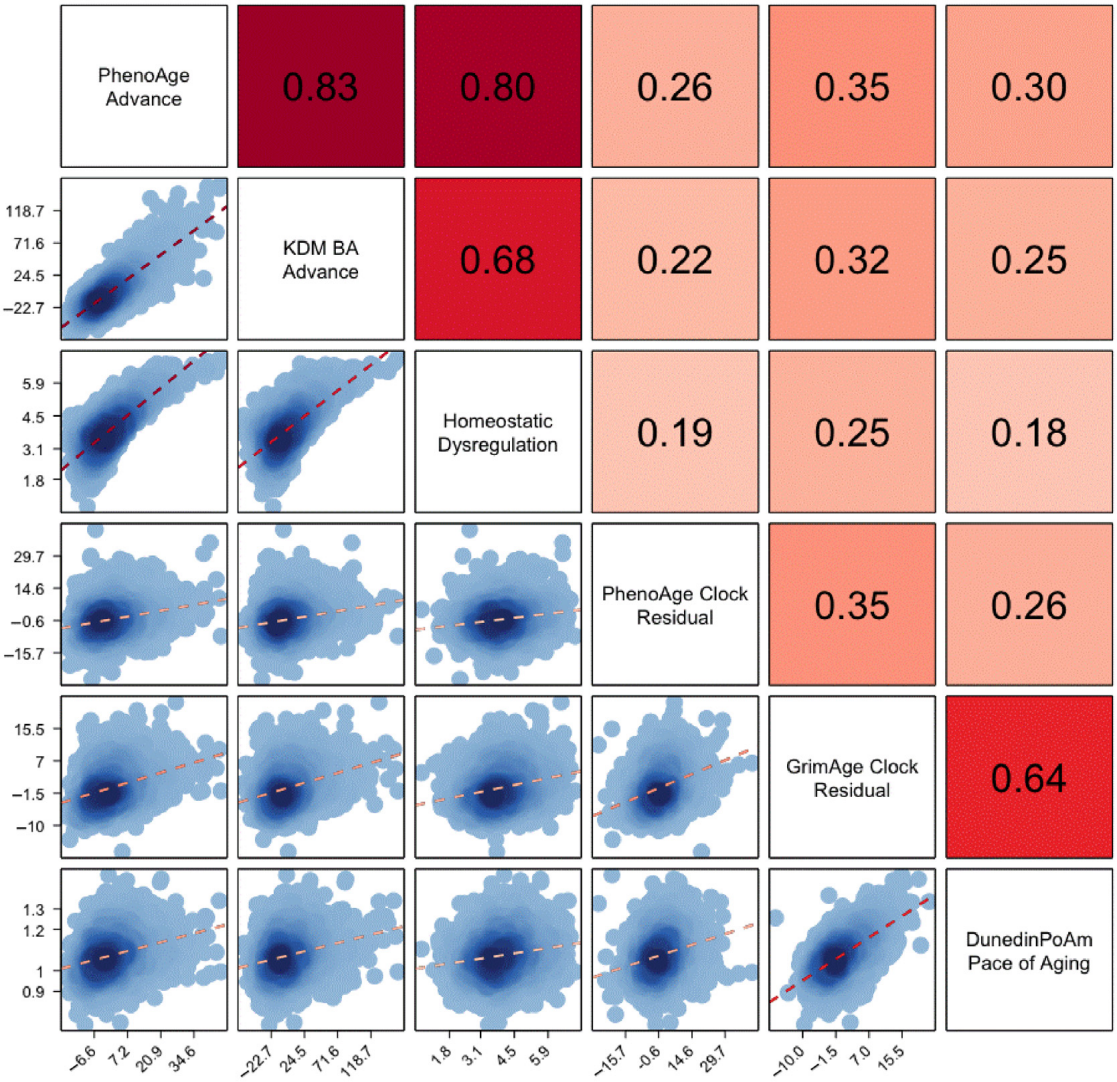
Correlations among 3 blood-chemistry and 3 DNAm measures of biological aging among Black and White participants in the US HRS. Biological aging measure labels are listed on the matrix diagonal. Pearson correlations are shown above the diagonal. Correlations are reported for the biological aging measures listed below and to the left of the cell. Scatterplots and linear fits illustrating associations are shown below the diagonal. The *Y-*axis of the plots correspond to the biological aging measure to the right of the cell. The *X-*axis of the plots correspond to the biological aging measure above the cell. Sample sizes for correlations among blood-chemistry measures are *n* = 9,255. Sample sizes for correlations between blood-chemistry and DNAm measures and among DNAm measures are *n* = 3,976.

Blood-chemistry measures of biological aging were derived using 3 published methods: Phenotypic Age (22), Klemera–Doubal Method (KDM) Biological Age (28), and Homeostatic Dysregulation (29) applied to clinical chemistries and complete blood count data from venous blood draws. Algorithm parameterization for the KDM biological age and homeostatic dysregulation measures was conducted using the NHANES III data. PhenoAge parameterization was taken directly from the published article by Levine et al. (22). All blood-chemistry measures were implemented in the HRS data using the BioAge R package (https://rdrr.io/github/dayoonkwon/BioAge/) (30). Blood-chemistry and DNAm measures were moderately correlated in our sample (Pearson's r range: 0.18–0.35, Fig. 1).

DNAm measures of biological aging were obtained from the HRS (18). We conducted analysis of 3 measures: the PhenoAge clock (22), the GrimAge clock (31), and the DunedinPoAm Pace of Aging (6).

We refer to individual differences in the measures of biological aging as reflecting more/less advanced biological aging in the case of the blood-chemistry measures and DNAm clocks, and as reflecting faster/slower aging in the case of the DunedinPoAm DNAm measure. The blood-chemistry measures and the DNAm clocks have similar interpretation: They quantify how much biological aging a person has experienced up to the time of measurement. For those whose clock-ages are older/younger than their chronological ages, biological aging is more/less “advanced” relative to expectation. In contrast, DunedinPoAm measures how rapidly a person has been aging over the recent past. Values above the benchmark range of 1 year of change per 12-months interval indicate “faster” biological aging, whereas values below 1 indicate “slower” biological aging. Measures are described in more detail in Table 1 and A1.

**Table 1.** Measures of biological aging included in analysis. The table reports the 6 measures of biological aging included in the analysis. For each measure, the table reports the criterion used to develop the measure and the interpretation of the measure's values. Criterion refers to the quantity the biological aging algorithm was developed to predict. Interpretation refers to the inference about biological aging that can be made on the basis of the values of the measure.

Measure		Criterion	Interpretation
**Blood-chemistry measures**. All algorithms were parameterized using data from NHANES III and included the following blood chemistries: albumin, alkaline phosphatase, creatinine, C-reactive protein (log), white blood cell count, lymphocyte %, mean cell volume, and red cell distribution width. PhenoAge additionally included glucose. KDM Biological Age and Homeostatic Dysregulation additionally included glycated hemoglobin (HbA1C). PhenoAge and KDM Biological Age algorithms included information about chronological age. For analysis, PhenoAge and KDM Biological Age were differenced from chronological age to calculate biological-age advancement values.			
	PhenoAge	Mortality	Age at which the participant's biomarker-predicted mortality risk matches the norm in the reference population (NHANES III).
	KDM Biological Age	Chronological Age	Age at which the participant's biomarker-predicted physiological integrity matches the norm in the reference population (NHANES III).
	Homeostatic Dysregulation	Deviation from healthy youth	Log biomarker-Mahalanobis distance of participant from young, healthy reference population (nonobese NHANES III participants aged 20–30 years).
**DNAm measures**. DNAm measures were developed from analysis of genome-wide DNAm measured on Illumina 27 k and 450 k arrays in a range of different datasets. The Horvath Clock was developed from the analysis of 82 different datasets. The Hannum Clock was developed from analysis of research volunteers at UC San Diego, University of Southern California, and West China Hospital. The PhenoAge Clock was developed from analysis of NHANES III and the InCHIANTI Study. The GrimAge clock was developed from analysis of the Framingham Heart Study Offspring Cohort. The DunedinPoAm Pace of Aging was developed from analysis of the Dunedin Study. DNAm measures were calculated by the HRS investigators. For analysis, DNAm clocks were residualized on chronological age to calculate biological-age advancement values.			
**Second generation DNAm clocks**			
	PhenoAge Clock	Blood-chemistry PhenoAge	DNAm prediction of the age at which the participant's biomarker-predicted mortality risk matches the norm in the NHANES III reference population (based on analysis of the InCHIANTI Study).
	GrimAge Clock	Mortality	Age at which the participants’ DNAm-predicted mortality risk matches the norm in the reference population (Framingham Heart Study Offspring cohort). The GrimAge clock was derived by first developing DNAm surrogates for blood proteins and smoking history, and then developing a mortality prediction model based on these DNAm surrogates, sex, and chronological age.
**Pace of aging**			
	DunedinPoAm Pace of Aging	Change over 12 years of follow-up in 18 system-integrity biomarkers	Years of physiological decline experienced per 1 year of calendar time over the recent past. DunedinPoAm was developed by modeling a composite of change scores for 18 biomarkers of organ system integrity from DNAm data. The expected value of DunedinPoAm in midlife adults is 1. Values > 1 indicate accelerated aging. Values < 1 indicate slowed aging.

#### Social mobility

We measured social mobility from participant reports about their socioeconomic circumstances before age 16, and from structured interviews about later-life wealth conducted by HRS between 1993 and 2016.

##### Childhood social origins

We constructed a childhood social origins index based on participants’ retrospective reports about their family's general financial circumstances relative to other families, their father's occupation, the family's experiences of economic hardship (family had to move due to financial difficulties, family received financial help, and father unemployed), and their parents’ educational attainment. We composed the childhood social origins index as follows: first, we conducted principal components analysis of financial circumstances, father's occupation, family economic hardship, and parents’ education scores for HRS participants with complete data on all items (*n* = 30,062). Second, we imputed missing values for father's occupation and parents’ education at the means for groups of participants matched on race, HRS birth cohort, and family financial circumstances score. Third, to compute the final factor scores, we multiplied the values of variables by their factor loadings from the complete-case principal component analysis and then averaged the products. Factor scores were computed for participants with nonmissing or imputed data on at least 3 of the 4 social origins variables (*n* = 37,620, of whom father's occupation was imputed for *n* = 4,279 and family educational attainment was imputed for *n* = 2,276). Additional details are reported in A2. For analysis, we converted factor scores to Z scores (M = 0 and SD = 1) and percentile ranks within 5-year birth cohorts. For the final childhood social origins index, higher values indicate more advantaged families of origin and lower values indicate less advantaged families of origin.

##### Later-life socioeconomic attainment

We measured later-life socioeconomic attainment from wealth data collected during structured interviews with participants about assets (including second homes) and liabilities over the course of multiple waves of participation in the HRS. Wealth data were chosen on the basis of evidence that wealth is more informative about social status in older adults as compared with income and educational level (32, 33), and shows clear associations with a range of aging-related health and functional deficits (34). We used wealth data compiled by the RAND Corporation (35) and merged with data distributed by the HRS. Because wealth data were combined across multiple years of measurement, we inflated all values to constant 2012 dollars. We applied an inverse-hyperbolic-sine transformation to reduce skew (36). Finally, we applied a theta transformation including adjustment for age and sex to achieve an approximately normal distribution of values (37). For analysis, we converted the transformed wealth values to Z scores (M = 0 and SD = 1) and percentile ranks to form later-life socioeconomic attainment scores. Higher values of the later-life socioeconomic attainment score indicate higher levels of attainment and lower values indicate lower levels of attainment.

##### Mobility

We measured social mobility from childhood to later-life using 2 complementary approaches. (1) Residualized-change: we regressed participants’ later-life-socioeconomic-attainment z-score on their childhood-social-origins z-score and calculated residual values as a measure of mobility. This approach quantifies mobility as the difference between the attainment a person achieved and the attainment expected based on their social origins. (2) Difference-score: we calculated mobility as the difference between the later-life socioeconomic attainment z-score and the childhood social origins z-score. This approach quantifies mobility as the absolute difference in rank between attainment and origins. These 2 measures of mobility were highly correlated (*r* = 0.76). We conducted parallel analysis of both measures. We also conducted analysis of social mobility measured in terms of percentile-rank change from childhood to later-life using both residualized-change and difference score approaches. Details of social mobility variables are reported in Table S1 (Supplementary Material; Panel B).

#### Disaggregating effects of mobility from effects of status attained through mobility

The effects quantified in our mobility analysis reflect combinations of the effects of the status attained through mobility and of mobility itself. Methods have been proposed to disaggregate these effects, although there remains no gold standard (38). A widely used method is the Diagonal Reference Model (DRM) first developed by Sobel (39, 40). The DRM estimates unique parameters to quantify the effects of status and the effects of mobility. DRM analysis is reported in A3.

#### Educational mobility

We conducted parallel analysis of mobility from participant reports about their own education and the education of their parents.

##### Parental education

We coded parental education in 3 categories based on years of schooling. To account for secular trends in educational attainment, we normalized parental educational attainments to 5-year birth cohorts of participants. We classified those with educational attainment below the 25th percentile as having low educational attainment, those with educational attainment between the 25th and 75th percentile as having average educational attainment, and those with educational attainment above the 75th percentile as having high educational attainment. We assigned the highest attainment category of either parent as the participant's parental educational attainment. This approach classified 20% of participants with low parental educational attainment, 57% with average parental educational attainment, and 23% with high parental educational attainment.

##### Participant education

We coded participant education into 3 categories: those who had not graduated from high school (22%), those who had graduated from high school but had not completed a college degree (53%), and those who had completed at least a college degree (25%).

##### Mobility

We calculated educational mobility as the difference in education categories between participants and their parents. We assigned index scores of 1, 2, and 3 to respondents’ educational attainment (less than high school, high school, and more than high school) and their parents’ educational attainment (low, medium, and high). We calculated educational mobility by subtracting parental education index scores from participant education index scores, such that negative values represent downward social mobility and positive values represent upward social mobility (range −2 to 2, mean = 0.02, and SD = 0.71). Details of educational mobility variables are reported in Table S1 (Supplementary Material; Panel C).

### Analysis

We used linear regression to test associations of social mobility with biological aging using the following specification:

$$\begin{equation*}
BA = \alpha + \beta *SE{S_T} + \gamma *X + \varepsilon,
\end{equation*}
$$

where $BA$ is the measure of biological aging, $SES$ is the socioeconomic circumstances measure (childhood social origins, later-life socioeconomic attainment, or social mobility), and$\,\,X$ is a matrix of covariates. All models included covariate adjustment for chronological age, specified as a quadratic term, sex, whether the respondent self-identified as Hispanic, self-identified race (White, Black, and other), and the interactions of age terms with sex, race, and Hispanic ethnicity. $\varepsilon $ represents the error term. The coefficient $\beta $ tests the association of the SES measure with biological aging. We report results for z-score transformations of mobility in the main text and report results for both metrics in the Supplemental Tables.

We tested if associations of social mobility with biological aging varied by childhood socioeconomic status, sex, or race by adding cross-product interaction terms to our models: BA = alpha + (beta*SES_T)+(delta*SES

$$\begin{equation*}
BA = \alpha + \beta *SE{S_T} + \delta *SE{S_T}*Z + \gamma *X + \mu + \varepsilon,
\end{equation*}
$$

where $BA$, $SES$, and *X* terms are the same as in the previous model and *Z* denotes the stratification variable (childhood socioeconomic position, sex, or Black/White racial identity). The coefficient $\delta $ tests the hypothesis that the association of mobility with biological aging varies across levels/strata of *Z*.

We used the same models to test associations of educational mobility with biological aging. In these models, the SES terms were replaced with terms for parents’ educational attainment, participants’ educational attainment, and the difference in attainments between parents and participants.

For all models, effect-sizes are scaled in standard deviation units of the outcome measure. Positive effect-sizes indicate more-advanced or faster biological aging; negative effect-sizes indicate less-advanced or slower biological aging. For social-mobility models, effect-sizes are reported for a 1 SD difference in the exposure. For educational mobility models, effect-sizes are reported for a single-category increases in educational attainment.

To account for nonindependence of observations of couples who share a household, we clustered standard errors at the household level. We conducted all analyses in RStudio Version 1.3.1093.

## Results

### Sample overview

HRS participants included in analysis showed substantial social mobility (percentile-rank mobility SD=25). Compared to the full 2016 HRS sample, participants in the VBS subsample and the DNAm subsample for whom biological aging measures could be computed were somewhat more likely to be White and to experience more upward social mobility. Comparison of socio-demographic characteristics of the analysis sample to the full 2016 HRS panel is reported in Table S1 (Supplementary Material) and Figure S4 (Supplementary Material).

### HRS participants who grew up in more socioeconomically advantaged households exhibited less-advanced and slower biological aging in later-life

**Fig. 2.**
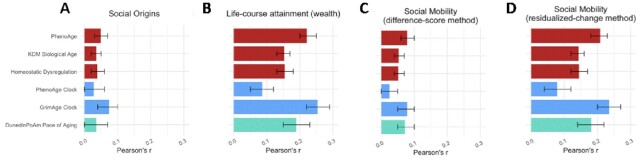
Effect-sizes for associations of childhood and later-life socioeconomic circumstances and social mobility with 6 blood-chemistry and DNAm measures of biological aging. The figure plots effect-sizes and 95% CIs from analysis of association between measures of social origins, social attainment, and social mobility with 6 measures of biological aging. Effect-sizes are reported in standard deviation units of the aging measures per standard deviation increment in the predictor, interpretable as Pearson's r. Blood-chemistry measures are shown in red (*n* = 9,255). 2nd generation DNAm clocks are shown in blue (*n* = 3,976). DunedinPoAm Pace of Aging is shown in turquoise (*n* = 3,976). All models are adjusted for age, sex, race, and Hispanic ethnicity.

We combined participants’ retrospective reports about their parents’ education, childhood experiences of economic hardship, and perceptions of their family's relative socioeconomic standing into a single index of childhood social origins. Participants who grew up in more socioeconomically advantaged households exhibited less-advanced and slower biological aging across all 6 aging measures included in our analysis (effect-size range $\beta $= [−0.07, −0.03], where ‘$\beta $’ represents an effect-size denominated in standard deviations of biological aging per standard deviation difference in social origins; Fig. 2a; Table S2, Supplementary Material). However, effect-sizes were small, consistent with a prior report from this cohort (21).

### HRS participants with higher levels of later-life socioeconomic attainment exhibited less-advanced and slower biological aging

We measured later-life socioeconomic attainment from household wealth data collected from structured interviews with participants about their assets and liabilities and compiled by RAND corporation. Participants with higher levels of attainment exhibited less-advanced and slower biological aging across all 6 measures of biological aging included in our analysis (attainment Z-score range $\beta $= [−0.25, −0.18], except for DNAm PhenoAge ($\beta $= −0.09), where ‘$\beta $’ represents an effect-size denominated in standard deviations of biological aging per standard deviation difference in attainment; Fig. 2b; Table S2, Supplementary Material). These effect-sizes were larger relative to the association of childhood social origins with biological aging.

### HRS participants who climbed up the social ladder showed less-advanced and slower biological aging in later-life

**Fig. 3.**
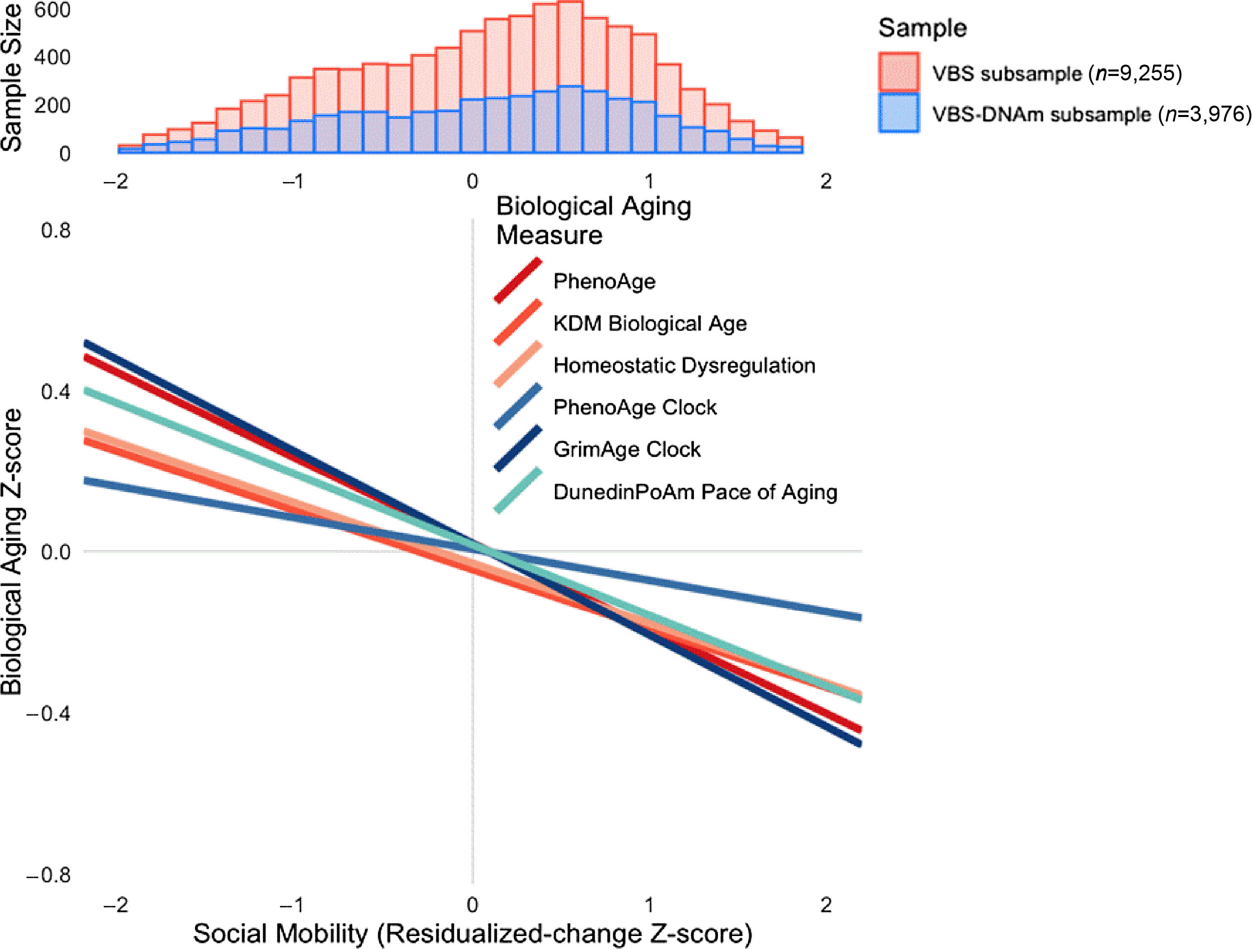
Effect-sizes for associations of life course social mobility with 3 blood-chemistry and 3 DNAm measures of biological aging. The histogram at the top of the figure shows the distribution of social mobility in percentile rank terms in the full HRS VBS (*n* = 9,255; red bars) and the DNAm subsample (*n* = 3,976; blue bars). The line plot at the bottom of the figure shows the association of social mobility with 6 measures of biological aging. Blood-chemistry-based measures are plotted in red shades. DNAm measures are plotted in blue shades. The figure shows that, across methods, upward social mobility was associated with less-advanced and slower biological aging.

We measured socioeconomic mobility in 2 ways. First, we computed mobility as the difference in the level of later-life socioeconomic attainment achieved from the level of attainment expected based on childhood social origins (the residual from a regression of later-life socioeconomic attainment on childhood social origins; hereafter “residualized-change mobility”). Participants with more upward mobility exhibited less-advanced and slower biological aging (residualized-change mobility Z-score range $\beta $= [−0.23, −0.16], except for DNAm PhenoAge ($\beta $= −0.09), where ‘$\beta $’ represents an effect-size denominated in standard deviations of biological aging per standard deviation difference in mobility). Second, we computed mobility as a simple difference score (later-life socioeconomic attainment—childhood social origins; hereafter “difference-score mobility”). Consistent with results from our first approach, participants with more upward mobility exhibited less-advanced and slower biological aging (difference-score mobility Z-score range $\beta $= [−0.09, −0.06] except for DNAm PhenoAge ($\beta $= −0.02)); Figs 2c, 2d; Fig. 3; Table S2, Supplementary Material).

#### Sensitivity analyses

We conducted sensitivity analyses to evaluate consistency of associations between social mobility and biological aging across 3 sets of groups facing different barriers to social mobility: those who grew up in more as compared with less disadvantaged families; women as compared with men; and Black as compared with White Americans.

##### Childhood social origins

The association between upward social mobility and biological aging was similar across the distribution of childhood social origins (interaction *P*-values > 0.237). This finding remained consistent when we restricted analysis to participants in the middle 50% of the childhood social origins distribution. Results are reported in Table S4 (Supplementary Material) and Figure S1 (Supplementary Material).

##### Sex

For both women and men, upward social mobility was associated with less-advanced and slower biological aging (for women, residualized-change mobility effect-size range $\beta $= [−0.26, −0.15] except for DNAm PhenoAge ($\beta $= −0.08), difference-score mobility effect-size range $\beta $= [−0.10, −0.05] except for DNAm PhenoAge ($\beta $= −0.004); for men, residualized-change mobility effect-size range $\beta $= [−0.28, −0.12] except for DNAm PhenoAge ($\beta $= −0.07), difference-score mobility effect-size range $\beta $= [−0.10, −0.04]). In residualized-change analysis, effect-sizes for blood-chemistry PhenoAge and Homeostatic Dysregulation measures of biological aging indicated somewhat stronger associations of mobility with biological aging for women as compared to men (interaction term range $\beta $= [−0.09, −0.04]). However, DNAm measures of aging did not show consistent differences, and effect-size differences were not generally statistically significant at the alpha = 0.05 level. In difference-score mobility analysis, effect-size differences between men and women were not statistically significant at the alpha = 0.05 level (*P* > 0.113). Results are reported in Table S5 (Supplementary Material) and Figure S2 (Supplementary Material).

##### Racial identity

For both White and Black adults, upward social mobility was associated with less-advanced and slower biological aging (for Black adults, residualized-change mobility effect-size range $\beta $= [−0.25, −0.16] except for DNAm PhenoAge ($\beta $= −0.09), difference-score mobility effect-size range $\beta $= [−0.11, −0.08] except for DNAm PhenoAge ($\beta $= −0.05); for White adults, residualized-change mobility effect-size range $\beta $= [−0.25, −0.15] except for DNAm PhenoAge ($\beta $= −0.09), difference-score mobility effect-size range $\beta $= [−0.09, −0.03]). Effect-size differences between White and Black adults were not statistically significant at the alpha = 0.05 level (*P*-values for tests of interaction > 0.052). Results are reported in Table S6 (Supplementary Material) and Figure S3 (Supplementary Material).

The consistency of effect-sizes for social-mobility associations with biological aging between White and Black HRS participants contrasts with reports that associations of socioeconomic attainment with health may be weaker for Black as compared to White Americans (14, 16, 17, 41). In these studies, socioeconomic attainment was measured from education. We, therefore, repeated our analysis with a mobility measure derived by comparing educational attainments of participants to those of their parents (hereafter, “educational mobility”).

#### Analysis of educational mobility

Effect-sizes for educational-mobility associations with biological aging were somewhat smaller than effect-sizes for social-mobility associations (range $\beta $= [−0.13, −0.02], Table S3 [Supplementary Material]). As in analysis of social mobility, blood-chemistry measures of biological aging indicated somewhat larger effect-sizes for women as compared to men (for women, effect-size range $\beta $= [−0.14, −0.06]; for men, effect-size range $\beta $= [−0.13,0.03]; Table S7 [Supplementary Material]). For Black and White adults, upward educational mobility was associated with less-advanced and slower biological aging (for Black adults, effect-size range $\beta $= [−0.20, −0.04]; for White adults, effect-size range $\beta $= [−0.17, −0.05]). Effect-sizes were smaller in Black as compared to White adults with the exception of DunedinPoAm analysis, which showed the reverse pattern. However, differences were not statistically significant at the alpha = 0.05 level in the DNAm sample. Results are reported in Table S8 (Supplementary Material).

## Discussion

We tested how life-course socioeconomic mobility related to healthy aging in a national sample of older adults in the United States. We measured healthy aging using blood-chemistry and DNAm measures of the state and pace of biological aging. There were 3 main findings. First, older adults who had grown up in socioeconomically at-risk families and those who had accumulated less wealth across their lives exhibited more-advanced and faster-paced biological aging as compared to those who grew up in more socioeconomically advantaged families. Second, those who overcame early-life disadvantage and climbed the social ladder to achieve upward mobility had less-advanced and slower-paced biological aging in later life as compared with those who were nonmobile or downwardly mobile. Third, upward-mobility associations with healthy aging were generally consistent for men and women, for White and Black adults, and for those who started life at different levels of socioeconomic position. In sum, we did not find evidence of net biological costs associated with the stresses of climbing the social ladder. Instead, findings suggest that upward socioeconomic mobility contributes to healthy aging, including in groups that face structural barriers to socioeconomic attainment.

Our findings were consistent across metrics of aging derived from different biological levels of analysis and developed using different models of the aging process. Childhood socioeconomic disadvantage, lower levels of wealth in later-life, and downward social mobility were each associated with more-advanced/faster biological aging across 3 blood-chemistry measures (blood-chemistry PhenoAge, KDM Biological Age, and Homeostatic Dysregulation) and 3 DNAm measures (PhenoAge Clock, GrimAge Clock, and DunedinPoAm Pace of Aging), although effect-sizes were smaller for the DNAm PhenoAge Clock. These 6 measures comprise biological clocks that estimate the extent of aging in a person (KDM Biological Age, the PhenoAge measures, and GrimAge), a measure of physiologic deviation from a healthy, youthful state (homeostatic dysregulation), and a Pace of Aging measure that estimates the ongoing rate of decline in system integrity (DunedinPoAm). Consistency of findings across biological levels of analysis and conceptually distinct measures of aging builds confidence in the robustness of the association of upward social mobility with healthy aging.

Our results contrast with some previous reports suggesting that there may be physical health costs from upward social mobility (14–17, 41). A possible explanation is that we measured life-course socioeconomic attainment from data on wealth accumulation, whereas previous studies had focused on educational attainment (14, 16, 17, 41). When we conducted analysis of educational mobility, our findings were more consistent with prior studies; effect-sizes for upward educational mobility were 2–4 times larger in analysis of White as compared to Black participants, with the exception of the DunedinPoAm Pace of Aging measure, for which the educational-mobility effect-size was larger in Black as compared to White participants. (In tests of interaction, effect-size differences were not statistically significant at the alpha = 0.05 level for any of the measures.)

The difference in findings in analysis of social mobility as compared to educational mobility may reflect differences in the life stage timing of the measurements used to quantify these processes and in the ways that the different mobility processes themselves affect the lives of Black and White Americans. The data we used to quantify life-course attainment in social mobility analysis was derived from structured interviews the HRS conducted with participants about their assets and liabilities during follow-ups spanning 1992–2016. These data capture levels of resource participants accumulated across their lives and had access to during the years leading up to the blood draws from which we derived our measures of healthy aging. Conversely, participants mostly completed their education decades before aging measurements were taken. Educational attainment plausibly represents young adult potential to accumulate socioeconomic and material resources that may affect healthy aging. However, this potential is likely unequally realized for Black and White Americans (42). An explanation for why educational mobility showed weaker associations with healthy aging in Black as compared to White participants is that Black Americans, who face racism in educational, work, and community environments, and who are part of extended family networks with lower levels of resources overall, do not realize the same social and material gains from their education as their White peers, e.g. (43, 44).

We acknowledge limitations. There is no gold standard measure of biological aging (5). Our conclusions are circumscribed by the precision and validity of available measurements. Our analysis included DNAm- and blood-chemistry-based measures. Other proposed levels of analysis for quantification of biological aging include proteomics, metabolomics, and physical performance tests. Ultimately, integrating information across levels of analysis may yield more precise measurements (45). However, consistency of results across different blood-chemistry and DNAm methods build confidence in findings. Social mobility was measured from participant-reported information. Reporting biases cannot be ruled out. Childhood socioeconomic circumstances, which were retrospectively reported, may be subject to recall bias. If aging trajectories affect recall of early-life adversity, or if participants’ anchoring their responses to different perceptions of normative socioeconomic conditions is related to other causes of aging, our findings may over- or under-estimate the true effects of social mobility on healthy aging. Studies are needed that can link measures of biological aging with administrative records that objectively record dimensions of social mobility. Our sample was made up of adults aged 50 years and older and their spouses. To the extent that socioeconomic disadvantage and downward mobility are associated with premature mortality, our sample may underrepresent the most at-risk population segments, potentially biasing our results toward the null. Further, mortality differences across demographic groups mean that differences between Black and White participants, and between men and women, may be underestimated. Participation biases may compound this survival bias, especially for Black–White comparisons; Black participants in the VBS were younger and healthier than the full sample of Black participants in the HRS (46). Our estimates of Black–White disparities are, therefore, likely to be conservative.

The observation that upward social mobility is associated with slower biological aging builds on evidence that people with more socioeconomic resources appear biologically younger than peers of the same chronological age with fewer socioeconomic resources (47). Mobility findings advance evidence for the hypothesis that intervention to promote economic well-being in adulthood can help to address disparities in healthy aging. But whether associations of upward mobility with slowed biological aging reflect effects of the resources acquired through upward mobility or from resources and characteristics that made mobility possible remains to be determined. A critical next step is to clarify when in the life course intervention can be most impactful and what mechanisms are most effective in delivering not just economic justice, but aging health equity. Collection of bio-samples from participants in studies of interventions to promote successful early-childhood development (48), increase educational attainment (49), and reduce poverty and promote stable housing and employment in adults (50, 51), can advance understanding of when and how interventions to address inequalities in social determinants of health can most powerfully affect inequalities in healthy aging.

## Funding

This research was supported by the National Institute on Aging (grants R01AG061378 and R01AG066887), the Russell Sage Foundation (grant 1810–08987), and the Jacobs Foundation.

## Authors' Contributions

GHG, YZ, and DWB designed the study, analyzed and interpreted the data, and wrote the first draft of the paper. GHG, YZ, BWD, KMH, MK, DK, PM, and DWB assisted with the analysis and interpretation of the data and the writing of the paper. GHG and DWB have had full access to all the data in the study and had complete freedom to direct its analysis and its reporting, without influence, editorial decision or censorship from the sponsors. The authors declare no competing interest and have all approved manuscript submission.

## Supplementary Material

pgac029_Supplemental_Files

## Data Availability

Data used in the study are available from the US Health and Retirement Study (https://hrs.isr.umich.edu/data-products). Analysis code is available on GitHub at https://github.com/gogogoglo/SocMob_BioAging.

## Notes

**Competing Interest:** D.W.B. is listed as an inventor on a Duke University and University of Otago invention that was licensed to a commercial entity.
