# Supplementary material for: Social mobility and biological aging among older adults in the United States
Source: PNAS Nexus. 2022 Mar 29;1(2):pgac029. doi: 10.1093/pnasnexus/pgac029 (PMC9123172; doi:10.1093/pnasnexus/pgac029)

Supplemental Methods

**Methods A1. Measures of Biological Aging.**

We selected three blood-chemistry measures of biological aging: Phenotypic Age (Levine et al., 2018), Klemera-Doubal Method (KDM) Biological Age (Klemera and Doubal, 2006), and Homeostatic Dysregulation (Cohen et al., 2013). The Phenotypic Age measure was developed from analysis of mortality risk; it represents the age at which a person’s blood-chemistry-predicted mortality risk would be approximately normal in the general population. The KDM Biological Age measure was developed from analysis of chronological age; it measures the age at which a person’s physiology would match the population norm. The Homeostatic Dysregulation measure was developed from analysis of deviation from a young, healthy reference sample; it quantifies how deviant a person’s physiology is from this reference.

We selected three DNA methylation measures of biological aging: the PhenoAge clock (Levine et al., 2018), the GrimAge clock (Lu et al., 2019), and the DunedinPoAm Pace of Aging measure (Belsky et al., 2020). The PhenoAge clock was developed from machine-learning analysis of the Phenotypic Age blood-chemistry measure. The GrimAge clock was developed in a two-stage analysis that first developed DNA methylation biomarkers of blood proteins and tobacco exposure and then fitted these DNA methylation biomarkers to mortality risk. Both the PhenoAge and GrimAge clocks measure the age at which a person’s mortality risk would be approximately normal in the population. DunedinPoAm was developed in a two-stage analysis that first developed a composite phenotype of within-person change over time in 18 biomarkers of organ system integrity, termed “Pace of Aging,” and then fitted DNA methylation data to predict that composite Pace of Aging .

**Methods A2. Variables included in the Social Origins Score.**

We analyzed social origins data for HRS participants with available data on race/ethnicity through the 2016 data collection wave (N=37,620).

Family financial circumstances were coded 1-3 based on responses of poor (coded as 1) about average (coded as 2), and pretty well off or variable (coded as 3).

Father’s occupation was coded 1-3 based on participant reports of their father working in manual occupations (coded as 1), clerical, service, and military occupations (coded as 2), and managerial/professional occupations (coded as 3).

The Family Economic Hardship Composite was coded 1-3 and was composed of three dichotomous indicators based on participants’ indications of whether their family had moved due to financial hardship, had received financial assistance, and if their father had been unemployed. Responses were summed to compute the composite score. In cases with missing data, the sum of non-missing values was divided by the number of non-missing values and the result was multiplied by three and rounded to the nearest integer to form the final composite score. Composite values were computed for participants with any non-missing data. The final composite was reversed for analysis such that a value of 3 corresponded to no economic hardship.

The Parents’ Education Composite was coded 1-3 and was computed in three steps. First, for each HRS birth cohort, we computed the 25^th^ and 75^th^ percentiles of years of education for participants’ mothers and fathers. Next, we recoded mothers’ and fathers’ years-of-education values according to the participant’s birth cohort (1 if the value fell below the 25^th^ percentile, if the value fell within the interquartile range, and 3 if the value exceeded the 75^th^ percentile). Finally, we assigned participants the highest education score of either parent.

Missing data on social origins variables are summarized in **Table A2.1.** Summary statistics of social origins variables are reported in **Table A2.2.**

**Table A2.1. Missing data on childhood social origins variables.**

**Table A2.2. Summary Statistics of childhood social origins variables.**

**Methods A3.**

Our primary mobility analysis aimed to address the question of whether upward socioeconomic mobility was associated healthier aging and if such an effect differed across social groups. We therefore focused our attention on the combined effects of the mobility process and the status attained through it. We refer to this combined effect as the *“total effect”* of mobility. The rationale for focus on this “*total effect*” is that it is the quantity most relevant for public health and social policy. Long-term benefit cost calculations for programs and policies to promote upward mobility include both the effects of mobility itself as well as the effects of ultimate attainment status, on healthy aging when quantifying benefits. Our difference-score- and residualized-change method analyses of mobility estimate these “*total effects*.”

However, there is a separate question of interest within research on social mobility about the effects of mobility itself, i.e., the specific effects of movement along the social gradient. For clarity, we refer to these effects as the *“marginal effects”* of mobility.

There are several proposed methods to quantify the marginal effects of mobility, with no agreed upon gold standard (1). A well-established method is the diagonal reference model (DRM), first proposed by Sobel (2, 3). The DRM estimates unique effects of status and mobility, isolating the *marginal effects* of mobility from the effects of origin and attainment statuses. To do this, the DRM involves two steps. In the first step, the DRM estimates the effects of status based on the subsample of individuals who are not mobile. Maximum likelihood estimation is used to define weights for the relative contribution of origins and attainments to the total status effect. In the second step, the DRM includes these status effects as covariates along with a series of additional variables that estimate the marginal effects of 1) being mobile (0/1), the direction of mobility (-1/0/1), and the magnitude of mobility (the number of categories across which a person transitions).

To contextualize our findings regarding the *total effects* of mobility, we conducted sensitivity analyses using the DRM to estimate *marginal effects* of mobility. The DRM requires status to be defined in discrete categories. To evaluate the stability of results, we conducted analyses based on categorization of status into tertiles, quintiles, and septiles of the social origins and attainments percentile-rank distributions. To maximize power for these analyses, we focus on the blood-chemistry PhenoAge measure. (This measure is correlated with the DNA methylation GrimAge Clock and DunedinPoAm pace of aging measures, shows similar effect-sizes to these measures in our primary analyses, and is available for roughly twice as many HRS participants.)

**Analysis.** Analysis was conducted using the Stata software (version 16). The DRM was fitted with the Stata package ‘drm’ (4). Models included the same covariates as our primary specifications (a quadratic specification of age, sex, race/ethnicity, and a set of interaction terms allowing the age effects to vary by sex and race/ethnicity).

**Results.** The proportions of participants in each mobility category and the mean level of biological aging (as measured by blood-chemistry PhenoAge-advancement Z-score) are shown in **Figure A3.1**. Results from DRM analysis are shown in **Table A3.1**. Marginal and total effects of mobility computed from these results are shown in **Figure A3.2**.

Tertile-based models estimated a 90/10 balance to the relative importance of attainment/origins in determining status effects. In contrast, quintile- and septile-based models estimated this balance at roughly 50/50. Mobility effects differed slightly between the models. In the tertiles-based model, the marginal effects of mobility were near zero. In contrast, in the quintiles- and septiles-based models, the marginal effects of mobility were larger and consistent in direction with upward mobility contributing to healthier aging and downward mobility contributing to less-healthy aging. In both the quintiles- and septiles-based models, the marginal effect of the magnitude of mobility was statistically different from zero at the alpha=0.05 level (p~0.02 in both models; **Table A3.1**).

To address the question of potential differential effects of mobility between Black and White Americans, we repeated DRM analysis in stratified samples of Black (n=1,562) and White (n=6,044) participants. We conducted stratified analysis using status quintiles defined in the full sample. Descriptive data for the Black and White samples are reported in **Figure A3.3**. DRM results for the Black and White samples are reported in **Table A3.2**. Marginal and total effects of mobility computed from these results are reported in **Figure A3.4**.

The DRM model for stratified samples of White and Black participants differed in the weights estimated for the contributions of origins and attainments to status effects (**Table A3.2**). Whereas the DRM for the White sample indicated that origins carried a greater weight as compared to attainments (weight=0.72 for origins as compared to 0.28 for attainments), the opposite was true of the DRM for the Black sample (weight=0.02 for origins as compared to 0.98 for attainments).

Mobility effects were also somewhat different by race (**Figure A3.4**). The overall magnitudes of marginal and total mobility effects were smaller in the Black-sample DRM as compared to the White-sample DRM. In addition, and the adverse effects of downward mobility, apparent for all downward moves in the White-sample, were apparent only for extreme downward moves in the Black-sample DRM.

There are limitations inherent to DRM models. DRM models require status to be defined in discrete categories. Results therefore depend on correct model specification; in our analyses, we saw significant variation in effect estimates and relative status weights based the number of categories into which status was divided. Results from three-category specification differed from results for the five- and seven-category specification. DRM models also require several assumptions, including that non-mobile individuals (those with identical origin and attainment statuses) are exchangeable with mobile individuals with regard to status effects, and that the relative contributions of origin and attainment to status effects are invariant across origin and attainment statuses and mobilities (1). These assumptions may not hold in highly-mobile samples (4,5).

**REFERENCES**

1. L. Luo, Heterogeneous Effects of Intergenerational Social Mobility: An Improved Method and New Evidence. *Am Sociol Rev*, 00031224211052028 (2021).

2. M. E. Sobel, Diagonal Mobility Models: A Substantively Motivated Class of Designs for the Analysis of Mobility Effects. *American Sociological Review* **46**, 893–906 (1981).

3. M. E. Sobel, Social Mobility and Fertility Revisited: Some New Models for the Analysis of the Mobility Effects Hypothesis. *American Sociological Review* **50**, 699–712 (1985).

4. C. Kaiser, DRM diagonal reference model stata (2018) https:/doi.org/10.17605/OSF.IO/KFDP6 (January 12, 2022).

5. Luo, L. Heterogenous Effects of Intergenerational Social Mobility: An Improved Method and New Evidence. (2021) https://osf.io/preprints/socarxiv/bsw7g/

**Table A3.1. Diagonal Reference Model (DRM) effect estimates.** The table shows status and mobility effect estimates for DRMs based on status tertiles (Panel A), quintiles (Panel B), and septiles (Panel C). Status effects represent the independent effects of occupying a status level for individuals who were immobile (i.e. who occupied the same tertile/quintile in childhood and adulthood). The model estimates the relative contribution of origin status and attainment status to the overall status effect in the terms ‘p’ and ‘q’. Mobility effects are divided into three variables, following Sobel’s original specification: a mover/stayer term indicating whether individual was mobile or immobile; a direction term, coding whether the individual was downwardly mobile, immobile, or upwardly mobile; and a steps term, coding the number and direction status transitions from origin status to attainment status. Models included covariates for a quadratic specification of age, sex, race/ethnicity, and a set of interaction terms allowing the age effects to vary by sex and race/ethnicity. Models included n=9,255 participants.

**Table A3.2. Diagonal Reference Model (DRM) effect estimates from stratified samples of White and Black participants.** The table shows status and mobility effect estimates for DRMs based on status quintiles for stratified samples of White (n=6,044, top panel) and Black (n=1,562, bottom panel) participants. Status quintiles were defined in the full HRS sample. Status effects represent the independent effects of occupying a status level for individuals who were immobile (i.e. who occupied the same tertile/quintile in childhood and adulthood). The model estimates the relative contribution of origin status and attainment status to the overall status effect in the terms ‘p’ and ‘q’. Mobility effects are divided into three variables, following Sobel’s original specification: a mover/stayer term indicating whether individual was mobile or immobile; a direction term, coding whether the individual was downwardly mobile, immobile, or upwardly mobile; and a steps term, coding the number and direction status transitions from origin status to attainment status. Models included covariates for a quadratic specification of age, sex, and a set of interaction terms allowing the age effects to vary by sex.

**Figure A3.1. Descriptive data for Diagonal Reference Model (DRM) Analysis.** The left column of matrices shows the proportions of the analysis sample (n=9,255) in each cell of the mobility matrix for tertile- (first row), quintile- (second row), and septile-based (third row) classifications of status. The right column of matrices shows the mean level of biological aging (blood-chemistry PhenoAge-advancement Z-score) for tertile-, quintile-, and septile-based classifications of status.

**Figure A3.2. Marginal and total effects of mobility estimated from diagonal reference models (DRMs).** The left column of matrices shows marginal-effects-of-mobility estimates computed from DRM mobility terms reported in Table S2.1 for each cell of the mobility matrix for tertile- (first row), quintile- (second row), and septile-based (third row) classifications of status. The right column of matrices shows total-effects-of-mobility estimates computed from DRM status and mobility terms reported in Table S2.1 for tertile-, quintile-, and septile-based classifications of status. The formula to compute marginal effects of mobility for upwardly mobile individuals was Mover/Stayer + Direction + Steps * mobility distance (i.e. the number of categories between origins and attainment). The formula to compute marginal effects of mobility for downwardly mobile individuals was Mover/Stayer - Direction - Steps * mobility distance. The formula to compute total effects of mobility combined the marginal effect of mobility with the status effects computed as Origin Status * p + Attainment Status * q.

**Figure A3.3. Descriptive data for Diagonal Reference Model (DRM) Analysis in stratified samples of White and Black participants.** The left column of matrices shows the proportions of participants in each cell of the mobility matrix for White (n=6,044, top row) and Black (n=1,562, bottom row) participants. The right column of matrices shows the mean level of biological aging (blood-chemistry PhenoAge-advancement Z-score) for White and Black participants.

**Figure A3.4. Marginal and total effects of mobility estimated from diagonal reference models (DRMs).** The left column of matrices shows marginal-effects-of-mobility estimates computed from DRM mobility terms (see Table S2.2) for each cell of the mobility matrix for White (n=6,044, top row) and Black (n=1,562, bottom row) participants. The right column of matrices shows total-effects-of-mobility estimates computed from DRM status and mobility terms (see Table S2.2) for White and Black participants.

Supplemental Tables and Figures

Table of Contents

**Table S1.** Demographic characteristics of HRS sample, VBS subsample, and DNAm subsample**2**

**Table S2**. Effect-sizes for associations of social origins, socioeconomic attainment, and social mobility with blood-chemistry and DNA-methylation measures of biological aging4

**Table S3.** Effect-sizes for associations of parental education, educational attainment, and educational mobility with blood-chemistry and DNA-methylation measures of biological aging**6**

**Table S4.** Test of difference in associations of social mobility with biological aging across levels of childhood socioeconomic status**7**

**Table S5.** Test of difference in associations of social mobility with biological aging between women and men**8**

**Table S6.** Test of difference in associations of social mobility with biological aging between Black and White participants**10**

**Table S7.** Test of difference in associations of educational mobility with biological aging between Women and Men**12**

**Table S8**. Test of difference in associations of educational mobility with biological aging between Black and White participants**13**

**Figure S1.** Associations of social mobility with blood-chemistry PhenoAge and DNA-methylation GrimAge and DunedinPoAm by social origins**15**

**Figure S2.** Associations of social mobility with blood-chemistry PhenoAge and DNA-methylation GrimAge and DunedinPoAm in men and women**16**

**Figure S3.** Associations of social mobility with blood-chemistry PhenoAge and DNA-methylation GrimAge and DunedinPoAm in Black and White participants**17**

**Figure S4.** Distribution of demographic and social mobility variables in the US Health and Retirement Study Venous Blood Study and its DNA methylation subsample**19**

**Table S1.** Comparison of demographic characteristics for participants in the 2016 wave of the US Health and Retirement Study and the subset of participants included in social mobility analysis. The full HRS sample consists of all participants in the 2016 Health and Retirement Study who provided demographic data and information on childhood socioeconomic status and household wealth (n=20607). The VBS-BA sample consists of all participants from the full HRS sample for whom biological-age values could be computed based on biomarker data obtained through the Venous Blood Study (n=9255). The VBS-DNAm sample consists of all participants from the full HRS sample for whom biological-age values could be computed based on biomarker data and DNA methylation data obtained through participation in the Venous Blood Study (n=3976). In Panel A, mean values for household wealth were calculated by first inflating wealth values to 2012 dollars using the Consumer Price Index and then taking the average across all HRS measurement waves. For analysis of mobility, wealth values were transformed according to the procedure described in the Methods section and converted to either Z-scores or percentile ranks. Residualized-change mean values reported in the table are not precisely equal to 1 for any of the samples because the regression to compute residuals included all HRS participants ever providing data on social origins and attainments (N=37,722). In Panel B, biological-age advancements were calculated by subtracting chronological age from biological age (BA-CA) for blood-chemistry measures. Age residuals were calculated by fitting a regression of biological age on chronological age to the full VBS-DNA Methylation Sample, then subtracting the fitted value from that estimated using DNA methylation clock calculations.

**Table S2. Effect-sizes for associations of social origins, socioeconomic attainment, and social mobility with blood-chemistry and DNA-methylation measures of biological aging.** The table reports effect-sizes for associations of childhood socioeconomic status (SES), adult attainment (household wealth), and social mobility with blood-chemistry and DNA methylation measures of biological aging. For Z-score measures, effect-sizes are denominated in standard deviation units of biological age advancement per standard-deviation increment in the predictor. For percentile-rank measures, effect-sizes are denominated in standard-deviation units of biological age advancement per 25-percentile-rank increments of the predictor.

**Table S3. Effect-sizes for associations of parental education, educational attainment, and educational mobility with blood-chemistry and DNA-methylation measures of biological aging.** Effect-sizes are denominated in standard-deviation units of biological aging per one-category increase in educational attainment/ mobility. Categories of participant education are <high school, high school graduate, college graduate. Categories of educational social origins (parental education) are defined by the 25th and 75th percentiles of years of education completed by parents of participants grouped into 5-year birth cohorts. The final panel of the table shows results for parental education and educational mobility based on coding of parental education by the degree criteria used to code participant education.

**Table S4. Test of difference in associations of social mobility with biological aging across levels of childhood socioeconomic status.** We tested differences in associations of social mobility with biological aging across levels of childhood SES by adding terms to our social mobility regression models for childhood SES level and the interaction of childhood SES level with mobility. The table reports coefficients for interaction terms from these models. Results are presented for the full HRS biomarker sample n=9255) and the HRS DNA methylation sample (n=3786), and for a subset of each sample comprised of participants in the middle 50% of the social origins distribution (HRS biomarker subsample n=4619, HRS DNA methylation subsample n=1890). The purpose of assessing the middle 50% of the social origins distribution was to ensure consistency of results among those whose mobility was not bounded at either end of the distribution.

**Table S5. Test of difference in associations of social mobility with biological aging between women and men.** We tested differences in associations of social mobility with biological aging between women and men by conducting stratified regression analysis and by adding a term to our social mobility regression models for the interaction of sex with mobility. The first set of columns report results from analysis of women. The second set of columns report results from analysis of men. The third set of columns report the test of difference in results between women and men. This test was conducted by pooling the samples of women and men and fitting the regression model with an additional product term testing the interaction of sex with the measure of social position/mobility. The coefficient reported in this column is the coefficient for the product term testing the interaction. For Z-score measures, effect-sizes are denominated in standard deviation units of biological age advancement per standard-deviation increment in the predictor. For percentile-rank measures, effect-sizes are denominated in standard-deviation units of biological age advancement per 25-percentile-rank increments of the predictor.

**Table S6. Test of difference in associations of social mobility with biological aging between Black and White participants.** We tested differences in associations of social mobility with biological aging between participants identifying as Black and White by conducting stratified regression analysis and by adding a term to our social mobility regression models for the interaction of racial identity with mobility. The first set of columns report results from analysis of women. The second set of columns report results from analysis of men. The third set of columns report the test of difference in results between women and men. This test was conducted by pooling the samples and fitting the regression model with an additional product term testing the interaction of racial identity with the measure of social position/mobility. The coefficient reported in this column is the coefficient for the product term testing the interaction. For Z-score measures, effect-sizes are denominated in standard deviation units of biological age advancement per standard-deviation increment in the predictor. For percentile-rank measures, effect-sizes are denominated in standard-deviation units of biological age advancement per 25-percentile-rank increments of the predictor.

**Table S7. Test of difference in associations of educational mobility with biological aging between Women and Men.** We tested

differences in associations of educational mobility with biological aging between participants identifying as Women and Men by conducting stratified regression analysis and by adding a term to our educational mobility regression models for the interaction of sex with mobility. The first set of columns report results from analysis of women. The second set of columns report results from analysis of men. The third set of columns report the test of difference in results between women and men. This test was conducted by pooling the samples and fitting the regression model with an additional product term testing the interaction of sex with the measure of social position/mobility. The coefficient reported in this column is the coefficient for the product term testing the interaction.

**Table S8. Test of difference in associations of educational mobility with biological aging between Black and White participants.** We tested differences in associations of educational mobility with biological aging between participants identifying as Black and White by conducting stratified regression analysis and by adding a term to our educational mobility regression models for the interaction of racial identity with mobility. The first set of columns report results from analysis of Black participants. The second set of columns report results from analysis of White participants. The third set of columns report the test of difference in results between Black and White participants. This test was conducted by pooling the samples and fitting the regression model with an additional product term testing the interaction of racial identity with the measure of social position/mobility. The coefficient reported in this column is the coefficient for the product term testing the interaction.

**Figure S1. Associations of social mobility with blood-chemistry PhenoAge and DNA-methylation GrimAge and DunedinPoAm by social origins.** The figure plots associations social mobility with selected biological aging measures (one blood chemistry clock, one DNA methylation clock, and one Pace of Aging measure). Data are plotted separately for participants who grew up in families with low (purple), middle (orange), and high (teal) socioeconomic status (SES).


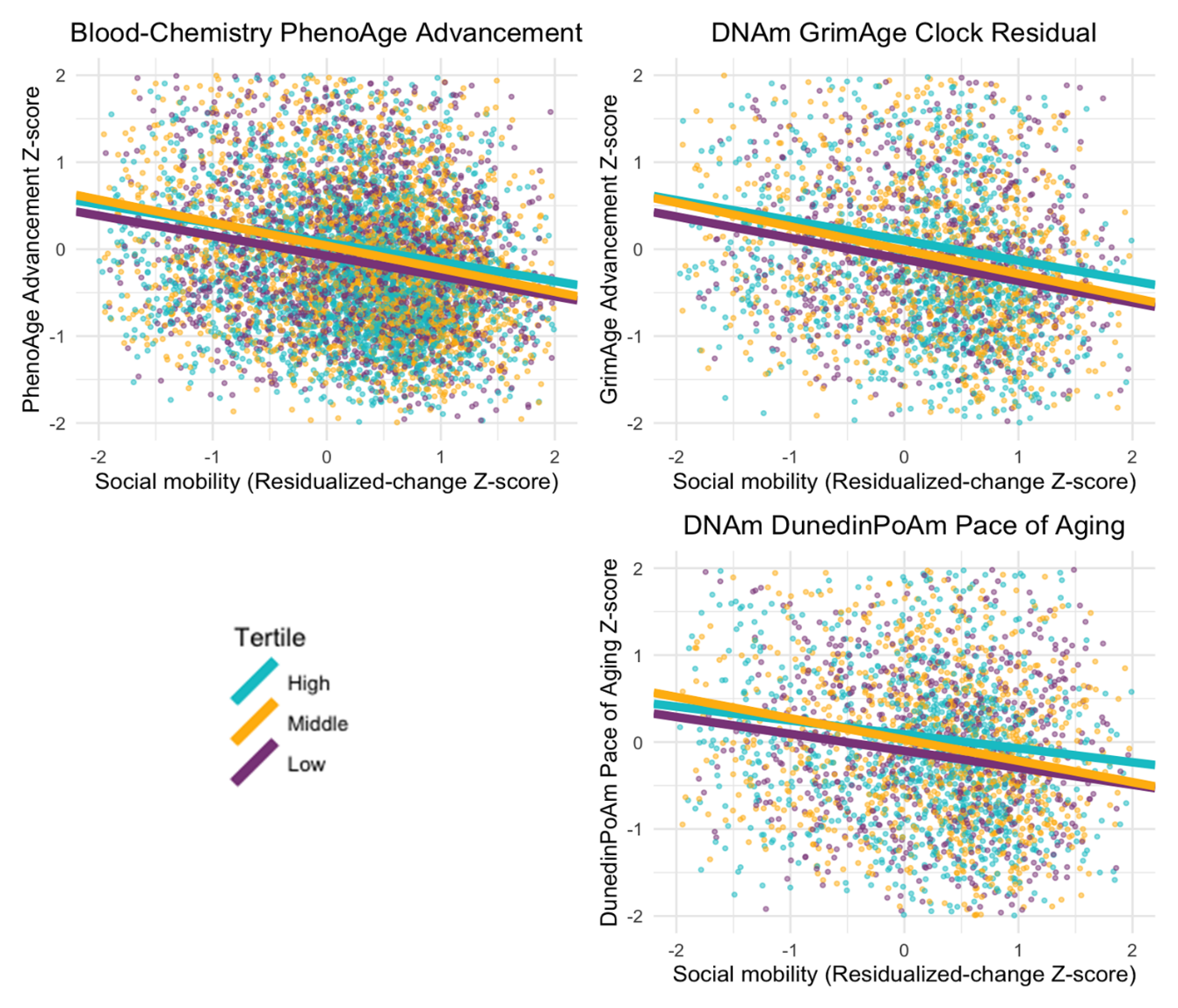


**Figure S2. Associations of social mobility with blood-chemistry PhenoAge and DNA-methylation GrimAge and DunedinPoAm in men and women.** The figure plots associations social mobility with selected biological aging measures (one blood chemistry clock, one DNA methylation clock, and one Pace of Aging measure). Data are plotted separately for men (orange) and women (green).


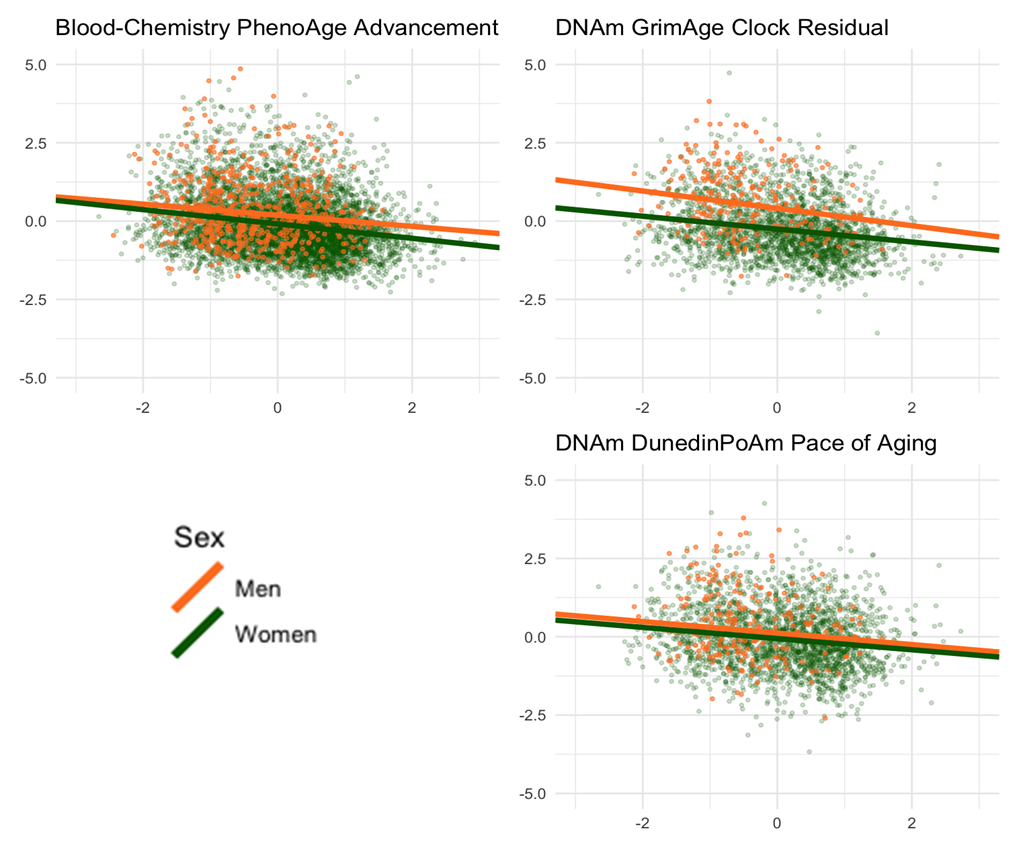


**Figure S3. Associations of social mobility with blood-chemistry PhenoAge and DNA-methylation GrimAge and DunedinPoAm in Black and White participants.** The figure plots associations social mobility with selected biological aging measures (one blood chemistry clock, one DNA methylation clock, and one Pace of Aging measure). Data are plotted separately for participants reporting Black (blue) and White (red) racial identity.

**
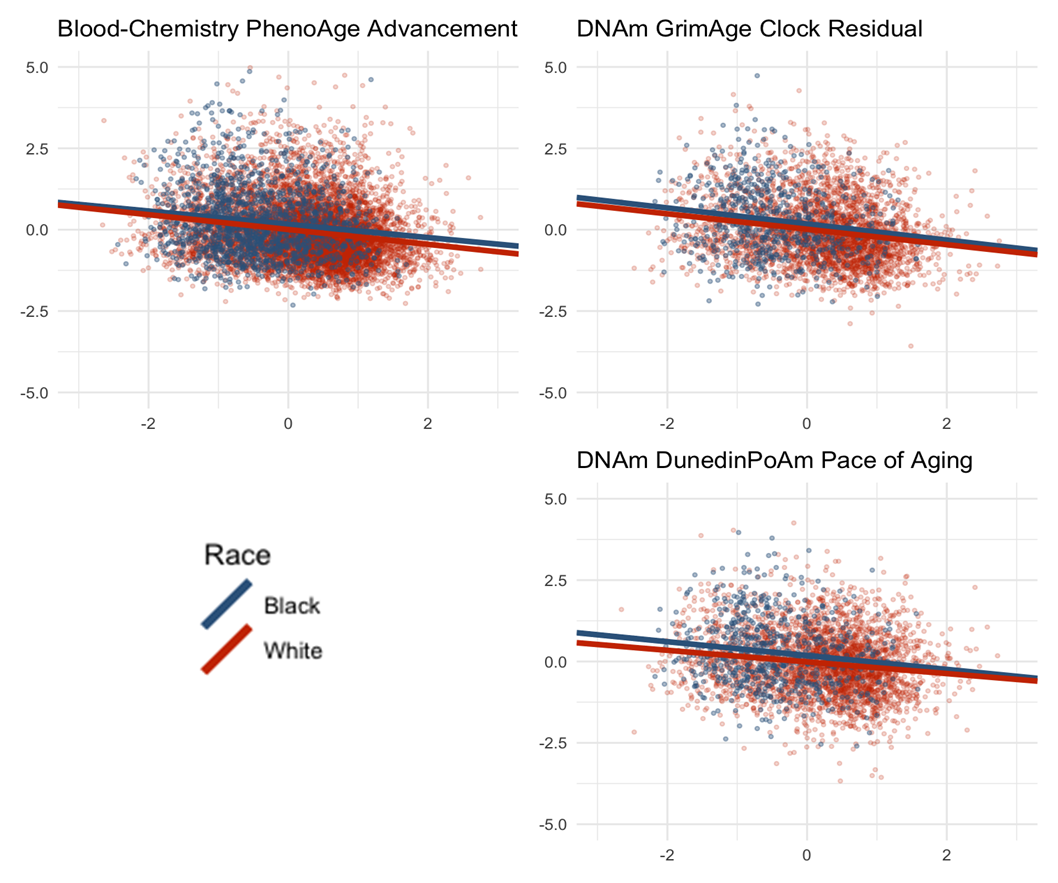
**

**Figure S4. Distribution of demographic and social mobility variables in the US Health and Retirement Study Venous Blood Study and its DNA methylation subsample.**


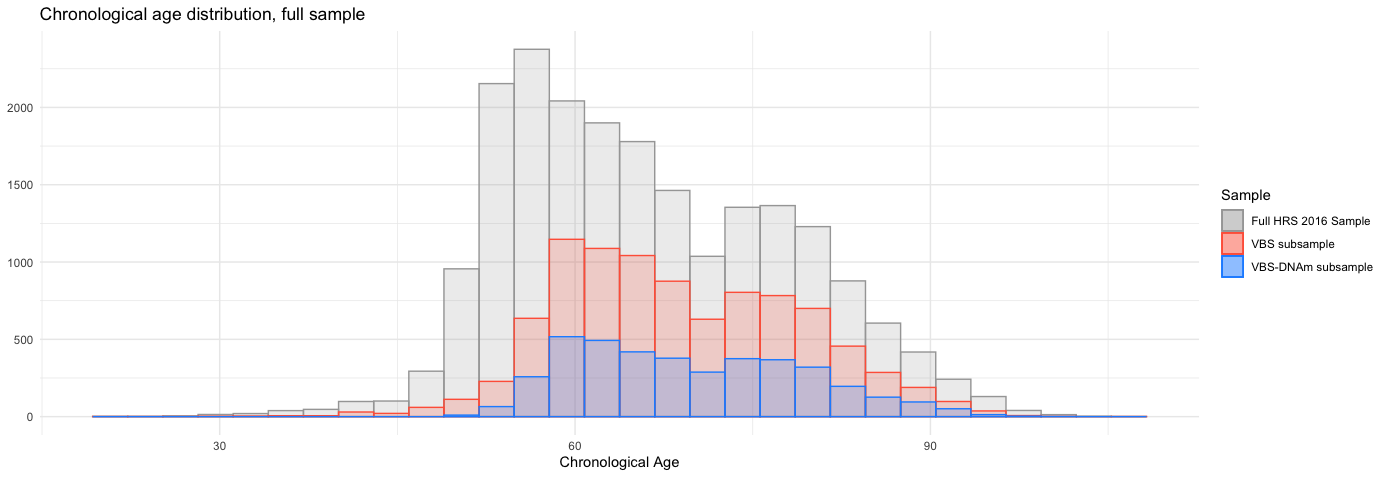


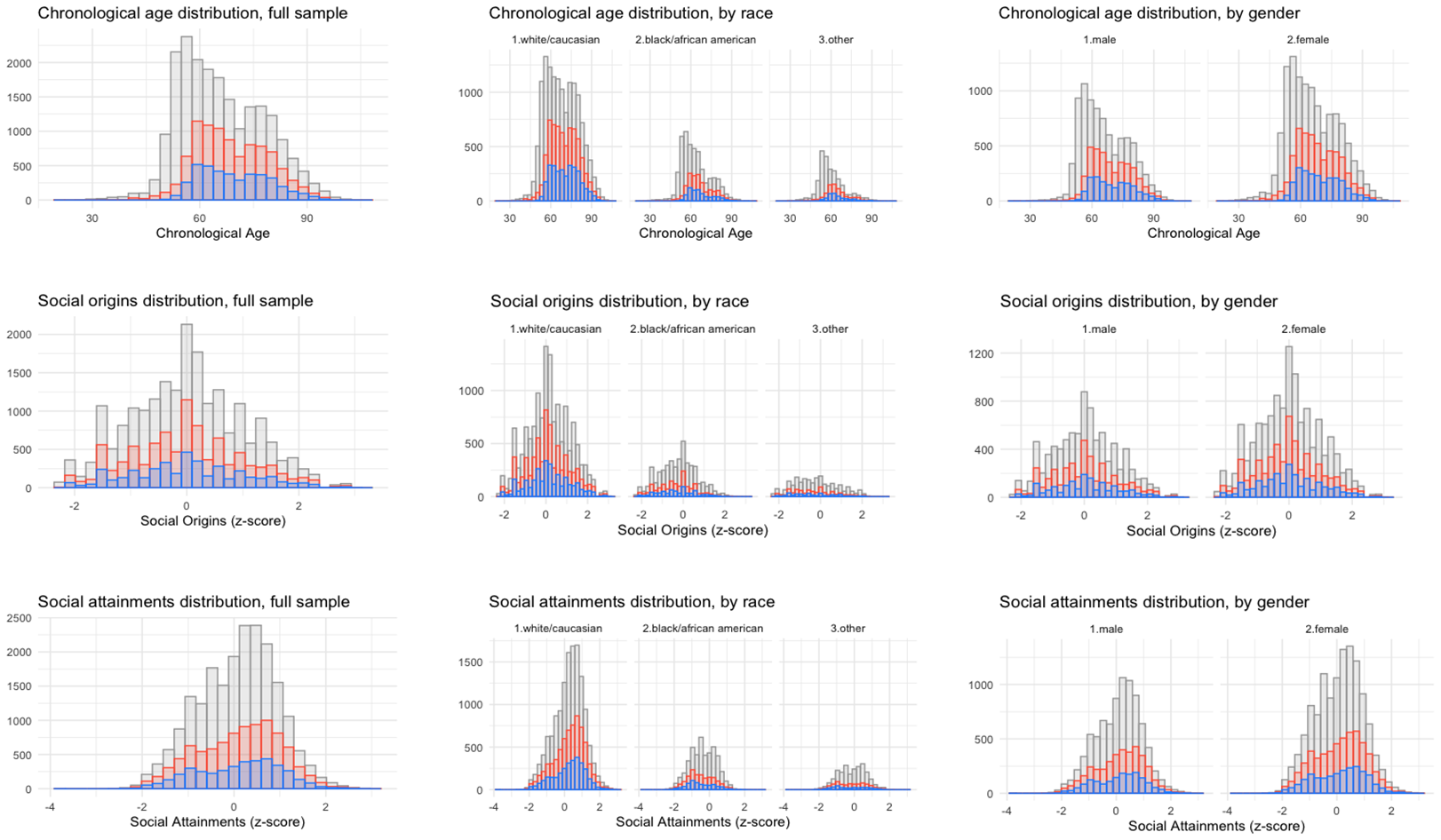


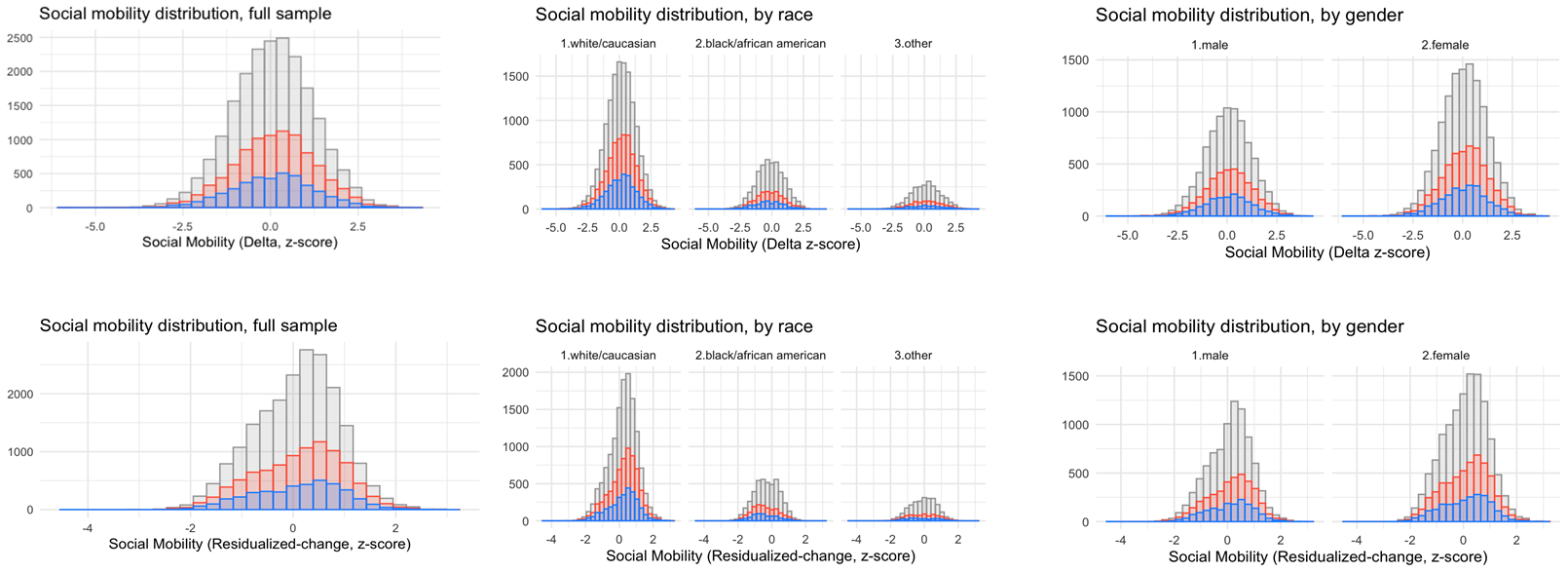

Supplement: pgac029_Supplemental_Files [file pgac029_supplemental_files.zip › PNASNEXUS-PNASNEXUS-2021-00133-file001.docx]
