# Supplementary figures and images for: Social mobility and biological aging among older adults in the United States
Source: PNAS Nexus. 2022 Mar 29;1(2):pgac029. doi: 10.1093/pnasnexus/pgac029 (PMC9123172; doi:10.1093/pnasnexus/pgac029)

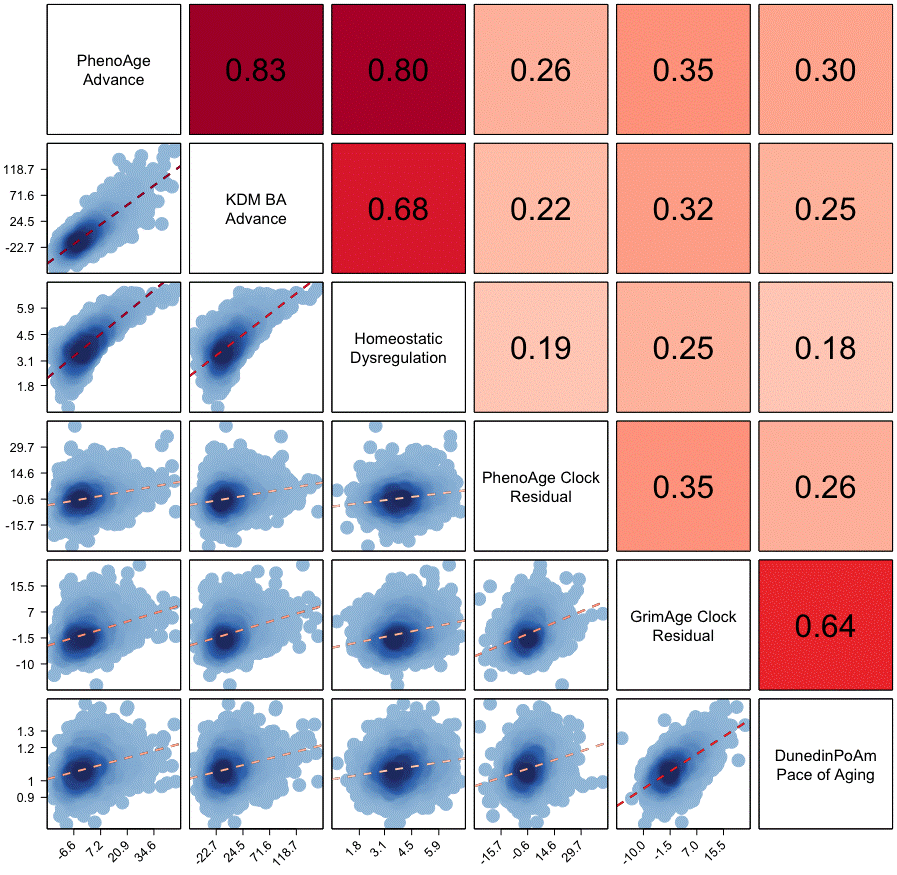

Supplement: pgac029_Supplemental_Files [file pgac029_supplemental_files.zip › PNASNEXUS-PNASNEXUS-2021-00133-s01.gif]
